# Cultural and health beliefs of pregnant women in Zambia regarding pregnancy and child birth

**DOI:** 10.4102/curationis.v38i1.1232

**Published:** 2015-04-10

**Authors:** Namakau C. M'soka, Langalibalele H. Mabuza, Deidre Pretorius

**Affiliations:** 1 Bupilo Family Health Clinic, Lusaka, Zambia; 2 2Department of Family Medicine, Sefako Makgatho Health Sciences University (formerly known as University of Limpopo, Medunsa), South Africa; 3 University of the Witwatersrand, Johannesburg, South Africa

## Abstract

**Background:**

Health beliefs related to pregnancy and childbirth exist in various cultures globally. Healthcare practitioners need to be aware of these beliefs so as to contextualise their practice in their communities.

**Objectives:**

To explore the health beliefs regarding pregnancy and childbirth of women attending the antenatal clinic at Chawama Health Center in Lusaka Zambia.

**Method:**

This was a descriptive, cross-sectional survey of women attending antenatal care (*n* = 294) who were selected by systematic sampling. A researcher-administered questionnaire was used for data collection.

**Results:**

Results indicated that women attending antenatal care at Chawama Clinic held certain beliefs relating to diet, behaviour and the use of medicinal herbs during pregnancy and post-delivery. The main beliefs on diet related to a balanced diet, eating of eggs, okra, bones, offal, sugar cane, alcohol consumption and salt intake. The main beliefs on behaviour related to commencement of antenatal care, daily activities, quarrels, bad rituals, infidelity and the use of condoms during pregnancy. The main beliefs on the use of medicinal herbs were on their use to expedite the delivery process, to assist in difficult deliveries and for body cleansing following a miscarriage.

**Conclusion:**

Women attending antenatal care at the Chawama Clinic hold a number of beliefs regarding pregnancy and childbirth. Those beliefs that are of benefit to the patients should be encouraged with scientific explanations, whilst those posing a health risk should be discouraged respectfully.

## Introduction

Health beliefs about childbirth are as old as human history itself (Gelis 1991:33–54). Globally, studies have indicated that there is often a conflict between the orthodox medicine and the traditional beliefs of women (Harris, Fleming & Harris 2012:41; Hoang, Le & Kilpatrick 2009:7). In developed countries, most births take place in hospitals or delivery centres, where the available nurses, midwives and obstetricians follow the biomedical model of practice. Furthermore, in these countries, pregnancy is seen as a medical condition which is largely treated using the biomedical model, with scientific explanations of procedures involved as well as the complications that may occur (O'Leary *et al*. 2007:861–862). However, as a result of globalisation, these developed countries frequently have community members from other cultures with health beliefs that need recognition and appropriate responses (Bodo & Gibson 1999:685; Hoang *et al*. 2009:10; Kim-Godwin 2003:77).

According to the World Health Organization (WHO), almost all maternal deaths (99%) occur in developing countries, with more than half occurring in sub-Saharan Africa (WHO 2013). Studies have revealed certain health beliefs that pose a danger to the health of the woman (Geloo 2003:2; Kyomuhendo 2003:23; Maimbolwa *et al*. 2003:37). In Tanzania, a study revealed increased mortality when women or their husbands followed traditional customs of administering traditional medicine to the woman during pregnancy and post-delivery (Evjen-Olsen *et al*. 2008:7). A study conducted in Uganda showed that there was poor utilisation of the available Western maternal services, as most women preferred to give birth on their own at home, using a relative, a traditional birth attendant or another unqualified practitioner. Maternal death was seen as a ‘sad but normal event’ (Kyomuhendo 2003:19–20). Traditional birth attendants were preferred because they did not ask for payment before attending to the women. They also respected traditional customs, for example, placental rites (Atuyambe *et al*. 2009:788–789, 793). In sub-Saharan Africa, studies have indicated that certain traditional practices and beliefs can pose a risk to the wellbeing of the mother and or child and may determine whether or not a woman will seek antenatal care (ANC) at a health facility (Kyomuhendo 2003:19; Maimbolwa *et al*. 2003:37). Although industrialisation and the advent of the biomedical model of health have led to a reduction of the influence of many of the health beliefs surrounding child birth (Cahill 2001:338), it has been shown that many women have to overcome a number of health beliefs which may act as barriers to following what is recommended or advised by healthcare practitioners (Maimbolwa *et al*. 2003:37).

In Zambia, Maimbolwa *et al*. (2003:38) studied the cultural childbirth practices and beliefs amongst the women who escorted pregnant women to the health facility during labour (*mbusas*). The *mbusas* were advisors to the women regarding appropriate traditional practices and childbirth. They encouraged pregnant women to use herbs as they believed that these helped to precipitate labour and widened the birth canal. Complications occurring during labour were attributed to witchcraft or were seen as punishment for misdeeds committed by the pregnant woman. In another study, women generally believed that they would be deprived of medical care if they divulged to a healthcare practitioner that they had visited a traditional healer before the consultation (Banda *et al*. 2007:125).

At Chawama Health Centre, the principal researcher frequently encountered health beliefs that were expressed by the attending women or their relatives. Harris *et al*. (2012:41) noted that health beliefs could ultimately affect health outcomes as they influence behaviour. Since health beliefs at the health centre related to diet, behaviour and the use of medicinal herbs during pregnancy and post-delivery, they could have a direct effect on both mother and baby. Therefore, a need was identified to elucidate and determine to what extent each belief was held.

### Problem statement

In 2010, the maternal mortality rate in Zambia was 440 deaths per 100 000 live births, whilst the infant mortality rate was 63 deaths/1000 live births. These figures were high compared to the global maternal mortality rate of 210 deaths/100 000 live births and the global infant mortality rate of 37.2 deaths/1000 live births in the same period (The World Bank 2012). Health beliefs about pregnancy and childbirth affect the woman's choice to use healthcare facilities and the acceptance of the advice given by healthcare practitioners at a facility (Harris *et al*. 2012:41). In other cultures in Russia, health beliefs about pregnancy and the biomedical model of practice have been integrated successfully, in that women are allowed to practise their traditional customs as long as they prove to have no detrimental effect on the health of either the mother or the baby (Callister *et al*. 2007:20). This indicates that healthcare practitioners have to be aware of the beliefs held and practised by pregnant women in the community and what can be done to address these in a way that enhances both cooperation and the wellbeing of the mother and baby. At the time of the study, as far as the authors of this article could establish, there were no studies in Zambia conducted on the health beliefs of women who attended ANC regarding pregnancy and child birth. The researchers, therefore, set out to conduct this study to explore the health beliefs of women attending ANC at Chawama Clinic in Zambia regarding pregnancy and childbirth.

From the problem statement above, the following research question arose: ‘What are the health beliefs of women attending ANC at Chawama Clinic in Lusaka, Zambia regarding pregnancy and childbirth?’

#### Significance of the study

Since a pregnant woman's belief can have an effect on her pregnancy and childbirth, knowledge gathered from this study has the potential to enhance the trust between women and midwives. Pregnant women will realise that midwives have an interest in their culture and wellbeing which may lead to open discussion on other health issues. Furthermore, the information gathered will assist midwives to encourage the positive beliefs and practices in pregnant woman whilst discouraging those that are harmful. Midwives will be more conscious about and have insight regarding the possibility of risk based on a pregnant woman's health beliefs.

#### Aims of the study

The objective was to determine the dietary beliefs related to pregnancy and childbirth, behaviours believed to affect pregnancy and childbirth and beliefs regarding the use of medicinal herbs during pregnancy, delivery and the puerperium.

## Research design

### Research approach and method

The study was a cross-sectional survey. The researchers chose this design to use descriptive and inferential statistics in order to describe the health beliefs of the pregnant women and the extent to which each one is held (Trochim 2006).

#### Population and sampling

At the time of study, an average of 1204 patients per month attended the Maternal and Child Health (MCH) services at the study site. Women 18 years and older were eligible for participation. Younger women, those with pregnancy-related complications, those who were mentally unstable and those who could not understand any of the local languages of communication were excluded. At a confidence level of 95% and a confidence interval of 5%, the sample size was 291 patients. The clinic service was provided on Monday to Friday (20 days in a month), consulting an average of 60 patients per day. Through systematic sampling, 15 patients were recruited per day, with every fourth patient being invited to participate in the study. If any patient declined participation, the next patient in the queue was motivated to take part in the study.

#### Materials

A researcher-administered questionnaire was used for data collection. It was based on the beliefs that had been expressed to the researcher by pregnant women during clinical encounters extending over a few years, as well as a literature search (Callister *et al*. 2007:20; Kim-Godwin 2003:77; Raven *et al*. 2007:5–10). The health beliefs related to three areas: (1) diet during pregnancy and post-delivery; (2) the behaviour of the pregnant woman; and (3) the use of herbs during pregnancy. The questionnaire, designed in English, was based on the three areas of health beliefs and was achieved with the assistance of the statistician and a linguistic expert from the University of Zambia. It was translated into the two dominant local languages (Nyanja and Bemba). The linguistics expert conducted back-translation of the questionnaire into English to verify the correctness of the meaning of each question.

#### Data collection

Data were collected over a four-week period, from 08 December 2009 to 05 January 2010 at the Chawama Clinic, situated on the outskirts of Lusaka, Zambia. The questionnaire was administered by two trained female research assistants to ensure consistency and to enhance inter-rater reliability. Each research assistant conducted an information session for each recruited respondent in a secluded cubicle next to the antenatal hall. They obtained written informed consent before commencing with data collection.

#### Data treatment

Data were entered into and analysed using the Statistical Package for Social Sciences (SPSS) version 16.0 statistical software (SPSS Inc., Chicago, IL 2007). Frequency tables and percentage distributions were used to describe the different variables.

## Results

### Demographic characteristics

A total of 294 questionnaires were completed by respondents with the baseline characteristics, as shown in Table 1. The age of respondents ranged from 18 to 44, with a mean age of 25.21 years. The majority of respondents (*n* = 279; 94.9%) were married and a further majority (*n* = 273; 92.9%) were unemployed. Twenty-seven tribes were represented by the respondents, most of whom were the Bemba (*n* = 86; 20.3%) and Tonga (*n* = 34; 11.6%) tribes.

**TABLE 1:** Participants’ baseline characteristics (*n* = 294).

Characteristic	Number	Percentage
**Age (years)**		
< 20	44	15
20–24	109	37
25–29	74	25
30–34	44	15
35–39	10	3.4
40–44	9	3.1
Missing	4	1.4
Total	294	100
**Marital status**		
Single	14	4.8
Married	279	94.9
Missing	1	0.3
Total	294	100
**Employment**		
Yes	19	6.5
No	273	92.9
Self employed	1	0.3
Missing	1	0.3
Total	294	100
**Education**		
None	13	4.4
Primary	114	38.8
Secondary	161	54.8
Tertiary	3	1.0
Missing	3	1.0
Total	294	100
**Tribe**		
Bemba	86	20.3
Tonga	34	11.6
Nyanja	27	8.5
Senga	22	7.5
Mambwe	19	6.5
Others	105	35.8
Missing	1	0.3
Total	294	100

### Health beliefs on diet during and post-delivery

Table 2 illustrates the high proportions of women who held beliefs in the importance of a balanced diet (*n* = 283; 96.2%), as well as the avoidance of salt intake by the mother until the cord stump of the baby has healed (*n* = 220; 74.9%). There was no marked difference in the proportions of women who held the belief that eating eggs during pregnancy could cause the baby to be born without hair, compared with those who did not hold that belief (*n* = 158, 53.8% versus *n* = 119, 40.5%, respectively). The same minor difference in proportions was seen regarding the belief that eating sugar cane during pregnancy caused the baby to have rough, dry and cracking skin compared with those who did not hold this belief (*n* = 133, 45.2% versus *n* = 146,49.6%, respectively). The belief in okra (*Abelmoschus esculentus*) causing the child to salivate excessively if the mother had ingested it during pregnancy was held by 106 (36.1%) of the women.

**TABLE 2:** Traditional beliefs on diet during pregnancy and post-delivery (*n* = 294)

Traditional belief	Agree *n* (%)	Neutral *n* (%)	Disagree *n* (%)	Missing data *n* (%)
Eating a balanced diet is important in pregnancy.	283 (96.2)	1 (0.3)	10 (3.4)	0 (0.0)
Eating eggs can cause a baby to be born with no hair.	158 (53.8)	16 (5.4)	119 (40.5)	1 (0.3)
Eating okra (*Abelmoschus esculentus)* can cause the child to drool a lot and have a lot of mucus.	106 (36.1)	31 (10.5)	157 (53.4)	0 (0.0)
Eating offal during pregnancy can lead to a tight cord around the neck during delivery.	71 (24.1)	40 (13.6)	183 (62.2)	0 (0.0)
Eating bones during pregnancy can lead to a high head and prolonged labour.	68 (23.1)	53 (18.0)	173 (58.8)	0 (0.0)
Eating sugar cane causes the baby to have rough dry cracked skin.	133 (45.2)	15 (5.1)	146 (49.6)	0 (0.0)
Drinking alcohol, including *chibuku*, will result in a difficult labour as the baby will be too big.	83 (28.2)	45 (15.3)	165 (56.2)	1 (0.3)
Certain foods (e.g. meat and vegetables) should be avoided soon after delivery as they cause severe lower abdominal pain.	49 (16.7)	28 (9.5)	212 (72.1)	5 (1.7)
Salt should be avoided until the cord stump of the baby has healed.	220 (74.9)	2 (0.7)	67 (22.8)	5 (1.7)

### Behaviour of the pregnant woman

Table 3 illustrates that 266 respondents (90.5%) agreed that it was important to begin ANC as soon as a woman knows she is pregnant. Two hundred and eleven (71.8%) agreed that pregnant women should continue with their normal daily activities. Quarrelling with people was believed to lead to adverse pregnancy outcomes by 200 (68.0%) of the participants. It was believed by 163 (89.5%) of the participants that obstructed labour could be caused by unfaithfulness on the part of either partner. One hundred and thirty-seven (80.6%) agreed that unfaithfulness by the woman could lead to the occurrence of convulsions at the time of delivery. One hundred and forty-six (49.6%) disagreed that using condoms could result in having a ‘weak’ child. Two hundred and eighteen (74.2%) believed that breastfeeding a baby in public in the presence of other mothers could cause that child to become ill. One hundred and ninety-three (68.1%) disagreed that eating with family members soon after delivery could make the family ill.

**TABLE 3:** Traditional beliefs on behaviour during pregnancy questions (*n* = 294)

Traditional belief	Agree*n* (%)	Neutral*n* (%)	Disagree*n* (%)	Missing data*n* (%)
Antenatal care should be started as soon as a woman knows she is pregnant.	266 (90.5)	8 (2.7)	18 (6.1)	2 (0.7)
Pregnant women should continue doing their normal daily activities during pregnancy.	211 (71.8)	11 (3.7)	63 (21.4)	9 (3.1)
Quarrels with people during pregnancy can lead to a bad pregnancy outcome.	200 (68.0)	18 (6.1)	76 (25.8)	0 (0.0)
Bad rituals can be performed with the soil where a pregnant woman has stepped (to harm her).	181 (61.6)	31 (10.5)	81 (27.5)	1 (0.3)
Unfaithfulness of the woman during pregnancy can cause fits at delivery.	237 (80.6)	14 (4.8)	39 (13.3)	4 (1.4)
Unfaithfulness (of man or woman) during pregnancy can cause obstructed labour.	263 (89.5)	8 (2.7)	20 (6.8)	3 (1.0)
Using condoms during pregnancy will lead to a weak baby.	83 (28.2)	60 (20.4)	146 (49.6)	5 (1.7)
Sexual intercourse in late pregnancy should be avoided as this may injure the baby.	133 (45.3)	23 (7.8)	133 (45.3)	5 (1.7)
Breast feeding a baby in public with other mothers may cause illness in that child.	218 (74.2)	8 (2.7)	64 (21.8)	4 (1.4)
Cooking in the first week after delivery delays the healing of the cord stump.	193 (65.6)	7 (2.4)	89 (30.2)	5 (1.7)
Eating with family members (husband or children) soon after delivery may make them ill.	68 (23.1)	22 (7.5)	193 (68.1)	6 (2.0)

### Use of herbs during pregnancy and post-delivery

One hundred and forty-seven of the participants (50%) disagreed that traditional herbs could make delivery faster once labour had started. One hundred and ninety-five (66.4%) agreed that herbs could assist in a difficult labour by shortening the labour period. Two hundred and thirteen (72.4%) agreed that cleansing with herbs after a miscarriage was necessary in order to prevent illness in the family.

### Other health beliefs

Table 4 shows responses to the open-ended question asking for additional beliefs that may have been omitted from the questionnaire. Forty-one participants (13.9%) indicated that they held additional beliefs that were not on the questionnaire. Twenty-five (8.5%) stated that coitus should be avoided after delivery. Eleven (3.7%) stated a belief that neighbours were not invited to sit around the fire with the family where there was a newly-delivered mother. Another belief stated by five (1.7%) of the respondents was that pregnant women should not stand in the doorway as this practice is believed to result in obstructed labour.

**TABLE 4:** Traditional beliefs in the use of herbs during pregnancy and post-delivery (*n* = 294)

Traditional belief	Agree*n* (%)	Neutral*n* (%)	Disagree*n* (%)	Missing data*n* (%)
Traditional herbs can help to make delivery faster when labour starts.	119 (40.4)	28 (9.5)	147 (50.0)	0 (0.0)
A difficult labour can be assisted with some traditional herbs.	195 (66.4)	23 (7.8)	73 (24.8)	0 (0.0)
Cleansing with herbs needs to be done after a miscarriage to prevent illness in the family.	213 (72.4)	11 (3.7)	66 (22.4)	4 (1.4)
**Other beliefs**				
Avoid intercourse after delivery.	25 (8.5)			
Fire should not be shared after delivery.	11 (3.7)	0 (0.0)	0 (0.0)	0 (0.0)
Standing in a doorway can lead to obstructed labour.	5 (1.7)			

## Ethical considerations

Ethical clearance was obtained from the Medunsa Research and Ethics Committee of the University of Limpopo – Medunsa Campus (clearance certificate number REC/M/20/2009:PG) and from the University of Zambia Research and Ethics Committee (reference number 014-06-08). Permission to conduct the study at the clinic was obtained from the District Medical Officer for Lusaka District. Participation in the study was voluntary.

### Potential benefits and hazards

The research assistants explained to the respondents that there were no potential risks in participating in the study. The potential benefits would be improved patient care through the understanding of the patients’ health beliefs, which could influence health policies. Respondents were assured of the availability of assistance from healthcare practitioners at the clinic on any health-related matter resulting from participation in the study.

### Informed consent

The study was explained to each respondent and those consenting to participate were then asked to fill in the written consent form. Respondents who declined participation were not penalised in any way and continued with their normal ANC visits. Participation in the study was voluntary and respondents were also free to withdraw from participation or answering further questions even when they had already given consent. The completed questionnaires were coded to conceal respondents’ identity, thus anonymity and confidentiality were maintained.

## Trustworthiness

### Reliability

The questionnaire was translated into two local languages and the translations back-translated from the local languages to English. In this way, correlation in meaning and understanding was assessed. The research assistants were trained on how to collect data so that all participants were asked questions in the same manner.

### Objectivity

The main researcher was not involved in the administration of the questionnaire and data analysis was done by a statistician not involved in the study.

### Validity

The questionnaire measured health beliefs as had been related anecdotally by women attending the antenatal clinic. Content validity was ensured by seeking confirmation of each health belief by other women on different days so as to understand each from various sources. The open-ended question eliciting other beliefs that did not form part of the list in the questionnaire ensured a comprehensive collection and assessment of the health beliefs.

## Discussion

The study demonstrated the various health beliefs regarding pregnancy and childbirth that were held by women attending MCH services at Chawama Clinic in Lusaka. These were based largely on diet during pregnancy and post-delivery, behaviour during pregnancy, as well as the use of herbs during pregnancy and post-delivery. Since health beliefs influence all sectors of community practices (Harris *et al.* 2012:41), including the health sector, it is important for healthcare practitioners to understand them. The researchers are of the opinion that awareness of the patients’ beliefs by healthcare practitioners will enable the latter to contextualise their practice within communities.

### Health beliefs on diet

Regarding the belief that eating eggs can cause a baby to be born without hair, it is of concern that almost a third of the women interviewed were of this opinion. The nutritional value of eggs in pregnancy as a source of protein, vitamins and minerals has been well established (Williamson 2006:41). There is need for healthcare workers to raise awareness against this belief for the benefit of the pregnant women and their offspring. However, where there are cultural sensitivities with respect to egg ingestion by pregnant women, it is the responsibility of the healthcare practitioner to explore culturally-acceptable alternatives.

Approximately one in three of the participants believed that ingesting okra during pregnancy caused excessive salivation of the child. Okra is a flowering plant in the mallow family, also known as *lady*’*s fingers*, *bhindi* or *gumbo* in many English-speaking countries. Its green seed pods are edible (Bender 2005:11). It is slimy when cooked, hence the association with salivation of the baby if the mother was ingesting it during pregnancy. However, pregnant women need to be educated regarding the fact that the pods are good nutrition: they are rich in pectin which acts as roughage in the gastro-intestinal system and also contain iron, calcium and vitamin A, which are necessary during pregnancy and post-delivery (Kumar *et al*. 2010:3592).

Although the women believed that women who drank alcohol during pregnancy would deliver large babies and experience prolonged labour, there is no scientific evidence that supports the belief. That being said, the healthcare practitioners have the responsibility to educate patients (in general) and pregnant women (more specifically) of the risks to the baby if alcohol is taken during pregnancy (Kelly *et al*. 2010:43–46). In addition, a study by Stade, Clark and D'Agostino (2004:17) has shown that alcohol should be avoided during pregnancy as there are no safe limits for the use of alcohol during this period.

Since almost three quarters of the respondents agreed with the belief that salt should be avoided during pregnancy, this needs to be addressed by healthcare practitioners. Salt is essential for the body to function normally, whether a woman is pregnant or not, since the biochemistry of nerve and muscle function depends on sodium and potassium (Stufflebeam 2008:3). However, it needs to be emphasised that salt should be taken in moderation rather than in excess (Appel *et al*. 2006:306), in which case it could lead to oedema. They should also be made aware of foods high in salt content, such as processed foods and most fast foods. Current evidence based on a systematic review of clinical trials regarding salt consumption during pregnancy indicates that it should ‘remain a matter of personal preference’ (Duley, Henderson-Smart & Meyer 2005:7).

It was noteworthy that regarding beliefs on diet during pregnancy, those who maintained a neutral position on each of the stated beliefs were below 20% and below 11% for beliefs regarding behaviour. The isolated finding that more than 20% of the women held a neutral position regarding the belief that using condoms during pregnancy led to a weak baby needs further exploration in order to establish possible reasons for this neutrality.

### Health beliefs on behaviour

The high percentage of agreement on prompt beginning of ANC, maintenance of normal activities during pregnancy and avoidance of quarrels during pregnancy for a good pregnancy outcome should be commended amongst the pregnant women. Early attendance for ANC ensures identification of obstetrical problems and enables prompt intervention in the interest of the wellbeing of both mother and child. The *Guidelines for Maternity Care in South Africa* recommend commencement of ANC as soon as the woman suspects pregnancy (Department of Health 2007:20). Activity during pregnancy is recommended, but the level of activity should decrease, especially toward term in a patient with a high risk profile: vaginal bleeding, dyspnoea on exertion, dizziness, headache, calf pain (to rule out thrombophlebitis), preterm labour and amniotic fluid leakage (Artal & O'Toole 2003:8).

The high proportion of women with the beliefs that quarrel during pregnancy and infidelity by either partner could lead to bad pregnancy outcomes could be viewed as beneficial to the wellbeing of mother and child. However, without reinforcing the beliefs, the healthcare practitioners should provide a scientific explanation of the advantage of avoiding emotional tensions as well as infidelity, not only during pregnancy but as a way of a healthy lifestyle. Infidelity exposes an individual to sexually-transmitted infections, including HIV (Adimora *et al*. 2002:322). Studies have shown that maternal emotions have a negative effect on the pregnancy and the baby, including the risk for spontaneous abortion, preterm labour and for having congenital malformations and child growth-retardation (microcephaly in particular) (Mulder *et al*. 2002:6; Van den Bergh *et al*. 2005:243–248) and can also negatively affect prenatal bonding between the mother and child (Schroth 2010:4).

The belief on the effect of using condoms during pregnancy was of great concern: about one in four respondents held the belief that using condoms during pregnancy could lead to a weak child, whilst only about half disagreed, and one in five were neutral on this belief. This means that a quarter of the women believed that using condoms was harmful and a fifth of them were undecided. This could have led to failure by these women to take advantage of condom protection against sexually-transmitted infections, including herpes simplex virus type 2 (HSV-2) and HIV (Casper & Wald 2002:12; Peltzer & Mlambo 2013:4–5; Sandøy *et al*. 2012:7).

### Health beliefs on the use of herbs

Whilst 50% of the participants in our study disagreed that herbs could assist a woman to deliver faster once labour had started, two-thirds of the participants agreed that herbs could assist in a difficult delivery. About three-quarters of the participants held the belief that herbs should be used for cleansing after a miscarriage. Further questioning should perhaps look at what it is that the women are being cleansed from. The belief in the use of ‘health’ herbs during pregnancy and the perinatal period is widespread on the African continent and in other parts of the world (Banda *et al*. 2007:125; Lans 2007:7; Van der Kooi & Theobald 2006:13). In his study, Lans (2007:7–8) suggested that analysis of herbs is necessary in order to ascertain their efficacy and dangers.

## Limitations of the study

The questionnaire did not establish any explanation for the beliefs held by the pregnant women. For example, regarding the belief that salt should be avoided until the baby's umbilical cord stump has healed, further enquiry could have been done to establish the underlying reasons. However, since the study was a survey, further enquiry could be left for qualitative research approaches to explore a deeper understanding of the phenomenon. There was a potential selection bias as a result of the selected study period. However, patients attending the clinic during this period could not be expected to have significantly different characteristics to those attending during any other time of the year. The questionnaires were only available in two dominant local languages in the region and participants who spoke neither were excluded from the study. This exclusion could have deprived the study of other important views.

## Recommendations

The health beliefs that are of benefit to the patients should be encouraged with scientific explanations. In the same way, those health beliefs which could pose health risk factors should be discouraged respectfully, also with scientific explanations. Further studies are required to establish deeper understanding of each health belief so as to enhance the appropriate response.

## Conclusion

The study has revealed the health beliefs held by women attending ANC at the Chawama Clinic. These related to diet, the behaviour of the pregnant woman and the use of herbs during pregnancy and post-delivery. Some have been found to promote the wellbeing of both mother and baby, for example, the belief that alcohol consumption during pregnancy will harm the baby; whilst others have been found to pose possible health risks to mother and baby's wellbeing, for example, the belief that using a condom during pregnancy leads to a weak baby.
